# Young adults’ perceptions of using wearables, social media and other technologies to detect worsening mental health: A qualitative study

**DOI:** 10.1371/journal.pone.0222655

**Published:** 2019-09-18

**Authors:** Lindsay H. Dewa, Mary Lavelle, Katy Pickles, Caroline Kalorkoti, Jack Jaques, Sofia Pappa, Paul Aylin

**Affiliations:** 1 School of Public Health, Imperial College London, London, United Kingdom; 2 NIHR Patient Safety Translational Research Centre, Imperial College London, London, United Kingdom; 3 The McPin Foundation, Young People’s Network, London, United Kingdom; 4 West London NHS Trust, London, United Kingdom; University of Toronto, CANADA

## Abstract

**Background:**

Technological interventions may help support and improve mental health. However young peoples’ perspectives on using different technologies to detect deteriorating mental health in those already diagnosed with a mental health condition is lacking. The study aim was to explore the perspectives of young patients on the feasibility and acceptability of using wearables, social media and technologies to detect mental health deterioration.

**Methods:**

The study was co-produced with young adults with past mental health difficulties. Semi-structured interviews were conducted with young adults with a severe mental health condition in a private room at a community mental health site. Data was triangulated by comparing codes and ideas across the two co-researchers and two researchers over two virtual meetings. Themes were finalised and presented in a thematic map.

**Results:**

Sixteen participants were interviewed (81% female). There were four main themes: dealing with mental health symptoms, signs of mental health deterioration, technology concerns and technological applications to identify worsening mental health. Wearables and mobile apps were considered acceptable and feasible to detect mental health deterioration in real-time if they could measure changes in sleep patterns, mood or activity levels as signs of deterioration. Getting help earlier was deemed essential particularly in reference to dissatisfaction with the current non-technological mental health services. However, patients identified issues to consider before implementation including practicality, safeguarding and patient preference.

**Conclusion:**

Wearables and mobile apps could be viable technological options to help detect deterioration in young people in order to intervene early and avoid delay in accessing mental health services. However, immediate action following detection is required for the patient to trust and use the intervention.

## Introduction

One in eight children and young people suffer from a mental health condition in the United Kingdom (UK). [1] Half of mental health conditions start before age 14 yet the majority go undetected or undiagnosed. [2,3] Those that are diagnosed are referred to community child and adolescent mental health service (CAMHS) but the transition to adult services at age 18 can be problematic [4] and add to an already challenging time. Therefore, this cohort is often more vulnerable to deteriorating mental health. Severe outcomes can include increased emergency attendances at hospital, higher frequency of self-harm and increased risk of suicide attempts and completions. [5] Early identification of deterioration and rapid response can significantly reduce these adverse events. [5] There are numerous interventions designed to recognise early onset or deterioration of serious mental illness. [6,7] These include self-reported, subjective assessments, family and friends noticing changes in mood and behaviour, or face-to face assessments with a general practitioner (GP) and/or mental health care professionals. However, up to 35% of young people who are experiencing mental health difficulties do not engage with healthcare services [8] for to a variety of reasons including lack of access.

Technology use is ubiquitous in society and may be a useful vehicle to identify signs of deterioration remotely, facilitating early intervention, before adverse events occur. [9] Technological interventions such as wearables (i.e. electronic devices that can be worn), mobile apps, social media and other online platforms are accessible for young people. [9,10] Indeed in the UK, 14.8% of 18–24 year olds are wearable users; 95% have a mobile phone and 95% use the internet to connect to social networking sites on daily basis [11]. Research exploring the application of these technologies to support the management of mental illness have reported mixed outcomes, depending on diagnosis, population and technology type. [12–17] For example, online social interventions (i.e. social networking and online support groups) and mobile applications are deemed effective in reducing depression and anxiety, but have been shown to be less effective for people with schizophrenia, albeit across limited studies. [12–17] Although adult patients (ages 18–65) perceive the use of technologies to help treat and monitor mental health conditions as acceptable, [18–24] there is a lack of evidence about the specific perspectives of young people (aged 18–25) with a mental health diagnosis who are transitioning through a critical phase in life. The study aim is to explore the i) acceptability and ii) feasibility of wearables, social media and other technologies to detect mental health deterioration in young people with a mental health diagnosis.

## Method

The qualitative study was guided by the recommended consolidated criteria for reporting qualitative research (COREQ) checklist (S1 Table). [25]

### Participants

A purposive sample of young people with a diagnosis of a severe mental health condition were recruited via West London NHS Trust database of patients under the care of a community mental health team for adults with severe and enduring mental health problems. These patients were approached between 1^st^ June and 2^nd^ August 2018 by a consultant psychiatrist (SP) in the first instance to inform them of the study and ask if they would be happy for a researcher (LD) to contact them. The researcher contacted those who agreed via telephone, explained the study to them and provided them with an information sheet and consent form to view. If participants wished to participate an interview date was arranged. Written informed consent was obtained from all patients prior to interview.

### Inclusion criteria

Participants were eligible to take part if they were 18–25 years old, had a diagnosed mental health condition, were fluent English speakers and could provide informed consent to participate.

### Exclusion criteria

Participants were ineligible: the psychiatrist deemed them too mentally unwell (i.e. experiencing an acute episode), or at risk of harm to themselves or others, they had insufficient command of the English language or were unable to provide informed consent to participate.

### Semi-structured interviews

Interviews were conducted face-to-face in a private room by an experienced researcher (LD) and two co-researchers (CK and JJ) (i.e. young person with experience of mental ill health who is a member of the research team) at one site at West London NHS Trust. Information sheets were given out again to refresh the participants’ mind and informed consent was obtained. Topic guides, co-produced with young people with past mental health difficulties, were structured to reflect six main sections: i) introductory questions (following reassurance that they are in a safe space), ii) use of and attitudes towards technologies in general (e.g. experiences, benefits and limitations of using wearables, social media, mobile apps, other technologies etc.), iii) experience of mental health deterioration (e.g. journey of when they were first unwell, how it was initially and subsequently detected, iv) use of technology in relation to mental health (e.g. acceptability) and v) use of technology to detect mental health deterioration (e.g. feasibility). Interviews were audio-recorded (one participant declined to be audio recorded and instead, the researcher (LD) produced handwritten notes) and continued until data saturation was reached. They were transcribed verbatim. Transcripts were imported into NVivo qualitative research software [26] to help facilitate qualitative data management.

### Patient and public involvement

We advertised on a third-party charity organisation newsletter (The McPin Foundation, putting lived experience at the forefront of the research) and Twitter by tweeting a short message about the opportunity for young people with mental health difficulties and experience of technologies (e.g. smart phones, social media and/or wearables such as an Apple Watch or Fitbit) to work on a qualitative project, using appropriate hashtags (#mentalhealth, #youngpeople, #socialmedia, #technology, #patientandpublicinvolvement) and link to the application form. Approximately 100 young people on a charity database (who had indicated they were happy to be contacted) were also emailed about the opportunity to be involved. After an internal selection process, seven young people were recruited (one from Twitter and six through The McPin Foundation) who then co-created the interview topic guide and co-designed the information sheet, consent form and protocol. Subsequent changes were made to the documentation (e.g. language was put in plain English). All seven people were asked which role they would like to take on going forward (e.g. project management, data collection and analysis or dissemination); everyone was assigned the role they requested because there was no conflict. Going forward three young people (referred to as co-researchers) were trained in conducting interviews and coding transcripts over two days. A working model (co-produced and verified by the co-researchers) of the best approach to conduct interviews with co-researchers was implemented. Firstly, two of the co-researchers shadowed the researcher (LD) separately during one interview (with the participant’s consent) and were given the opportunity to ask questions. They then conducted two interviews each and were debriefed by the researcher (LD) after each interview about what went well and what they could improve on. The researcher (LD) conducted the remaining interviews. They subsequently coded three transcripts each (their own transcripts when possible); these codes were incorporated in our final themes. All co-researchers were paid in accordance with guidance. [27]

### Analysis

Demographic and clinical characteristics were obtained from patient medical records and reported. Inductive thematic analysis was conducted and guided by Braun and Clarke’s steps. [28] After familiarisation, the data were triangulated, by comparing codes and ideas from three transcripts across two independent researchers (LD and ML) and two independent co-researchers (CK and JJ) in a 2-hour virtual meeting (i.e. Skype). This approach helped to ensure reliability and quality of the findings. We subsequently co-created an initial framework to code the remaining transcripts throughout the data collection period to ensure data saturation was obtained. The two independent researchers (LD and ML) then coded five transcripts each and met to confer and agree on any new codes and subsequent themes over three sequential face-to-face meetings. The coding comparison query in Nvivo was then applied across the researchers to ensure agreement in placement before proceeding with the rest of analysis. The group (CK, JJ, LD and ML) met for another 2-hour virtual meeting to finalise theme names and create a thematic map. Several themes were changed, consolidated and better linked as a result of this meeting.

### Ethics statement

The authors assert that all procedures contributing to this work comply with the ethical standards of the relevant national and institutional committees on human experimentation and with the Helsinki Declaration of 1975, as revised in 2008. All procedures involving human subjects/patients were approved by Social Care Research Ethics Committee (18/IEC08/0004).

## Results

Sixteen participants took part in the study (81% female) and had a mean age of 22 ± 1.8, (range 19–24) (Table 1). Interviews lasted between 48 and 112 minutes, and in six interviews two interviewers were present: the researcher and the co-researcher. The participants had varying experiences of using technologies including social media (e.g. Facebook, Twitter, Instagram, WhatsApp, Snapchat); mobile apps (e.g. MyFitnessPal, Secret Zen, Headspace, Calm); wearables (e.g. Fitbits, Apple Watches) and others (e.g. blogging websites, Amino, YouTube). Primary mental health diagnoses varied but mainly included mood disorders (see Table 1) that were comorbid with other conditions (e.g. personality disorders). Coding agreement on initial sub-themes between the two independent researchers (LD and ML) was good (93%).

**Table 1:** Demographic details (n = 16).

Participants	N (%)
***Gender***	
**Female**	13 (81%)
**Male**	3 (19%)
***Primary mental health condition***	
**Bipolar or bipolar affective disorder with or without psychotic symptoms**	5 (31%)
**Depressive disorder**	5 (31%)
**Emotional Unstable personality disorder**	4 (25%)
**Other (Post Traumatic Stress Disorder, Attention Deficit Hyperactive Disorder)**	2 (13%)

**Fig 1:**
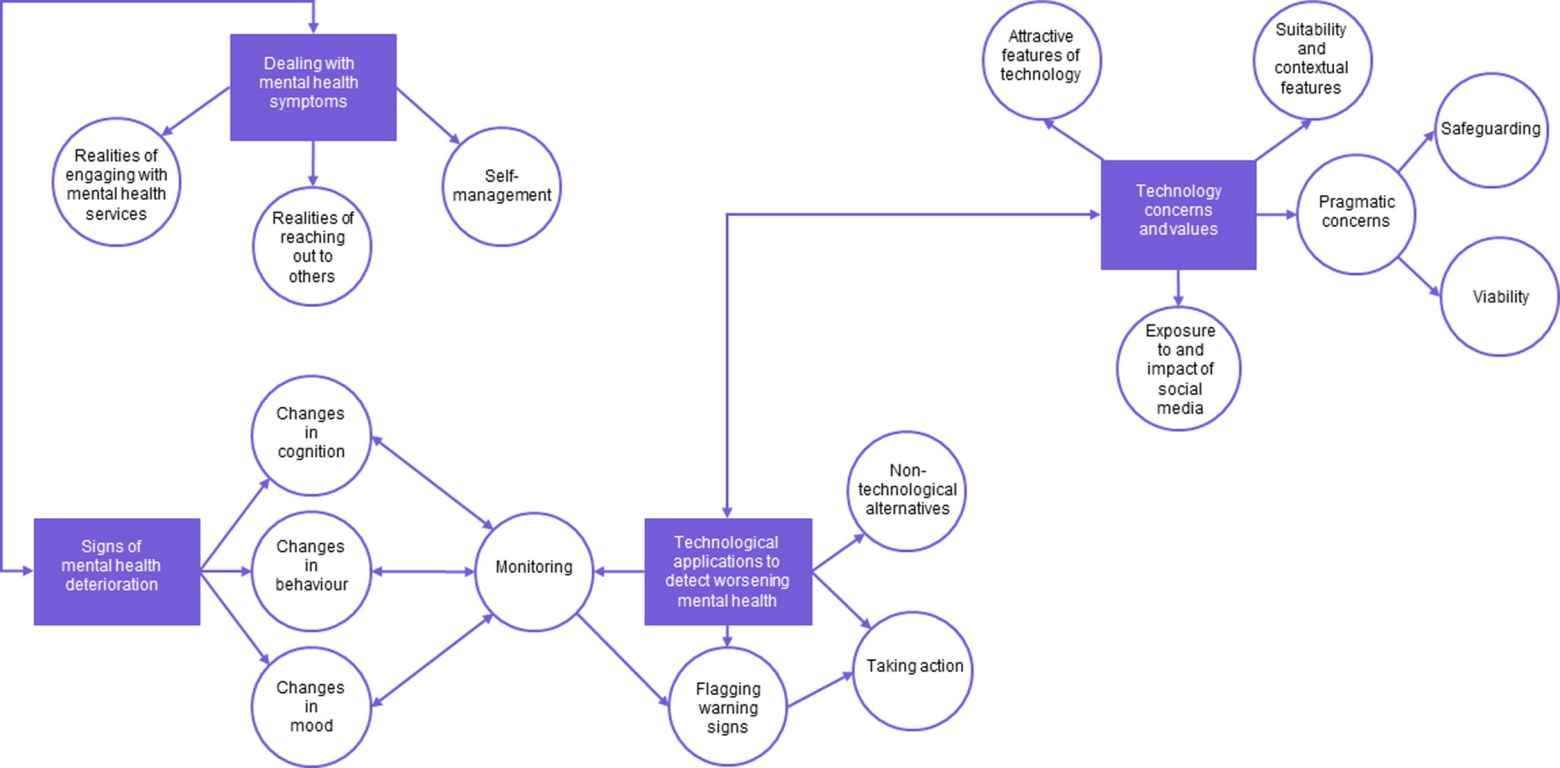
Thematic map.

There were four main themes: (1) dealing with mental health symptoms, (2) signs of mental health deterioration, (3) technology concerns and values and (4) technological applications to identify worsening mental health. A graphical representation of the relationships between themes is displayed in Fig 1.

### 1. Dealing with mental health symptoms

All participants discussed their mental health journeys: from when they were first unwell, to how their deterioration was first detected, to how they managed their mental health day-to-day. Their journeys centred on their experiences of getting help, whether it was from mental health services, other people (e.g. friends or family) or themselves.

#### Realities of engaging with mental health services

Half of the participants reported engaging with mental health services had been difficult at times and several participants reported bad experiences in accessing them at times of crisis when they particularly needed help. Similarly, some reported on the lengthy process to access care, and that there was no point in asking for help because it was not received.

*“…the mental health [services] is just absolutely 100% such a mess that…what’s the point in going to the doctor and saying you’re in crisis*, *because you’re just going to be in a waiting list for six months*.*”* (ID 9, Female)

*“When things got dramatically worse when I was…18…because it was escalated by the fact that I got discharged from CAMHS [Children and Adolescent Mental Health Services] but didn’t get handed over to anyone*. *I wasn’t okay…but they didn’t hand me over–I’m not sure why–but…said*, *‘Your doctor can handle*, *your GP [general practitioner]*, *and if things are really bad*, *go to A&E [accident and emergency services]*.*’ They just said that*, *and then within…a month… of when I was… starting school… after summer…things got worse again and I got referred to the eating disorder service in…the early September and that referral didn’t come through until November when…coincidentally*, *the day after I’d been in A&E [accident and emergency services] and they’d been… trying to admit me…Everything takes so long until they’re… ‘Oh*, *shit*, *we have to do something otherwise they’re going to die”* (ID 1, Female)

Furthermore, participants articulated the difficulty in accessing mental health services when they’re ill. For example, some participants indicated the need to be at a “certain level” of capacity to get in touch with services in the first place and that after getting in contact, the services do not make it easy to access the help they needed.

In contrast, a few participants had a good experience of accessing mental health services. For example, they mentioned that their mental health had improved after accessing the right type of support for them at the right time (e.g. a physical hug, talking it through, healthcare professional).

#### Realities of reaching out to others

Overall, experiences accessing help from other parties such as friends and family were reported as mixed. For example, most participants discussed the problems they had faced in opening-up to family and friends about their mental illness. Some reported they did not reach out because of the perceived stigma they would face or more commonly because they felt others would not understand.

*“I didn’t have any way of talking to my family about what I wanted or why I was feeling like that and they got quite*, *not judgemental*, *but they didn’t understand”* (ID 25, Female)

However, some participants indicated once they had opened-up and got through the initial hurdle, they then received the support they needed. Friends were commonly discussed as being supportive, capable of detecting mental health deterioration, and know when to intervene. For instance, one participant reflected on her experience when her friend noticed that her mental health had deteriorated and she needed help.

*“The police were called*, *my friends noticed that I was out of character*, *and I needed help*. *They rang the police and they rang an ambulance and I had to be handcuffed*, *strapped down and such and I was sectioned under 136*. *You know*, *put in seclusion in a hospital*. *But at that point I didn’t think I needed help*, *so my friends could see that I was in a crisis so they rang… the ambulance*, *and… my mental health team*. *And you now*, *a psychiatric nurse came and you know*, *reviewed how I was acting and… stuff like that”* (ID 9, Female)

#### Self-management

Most participants also discussed how they personally managed their mental health through i) being proactive and self-aware of their triggers, ii) using avoiding strategies and consciously hiding symptoms, and iii) using detrimental (unsafe) coping strategies. Seven participants indicated that over time they’ve become self-aware of when they are getting worse and what actions to take. Resulting positive actions included understanding what they needed and doing it (e.g. spending time alone, having a coffee), avoiding triggers, and going to hospital.

*“More recently when I went into hospital I knew that I needed to go in… I could have just gone to bed or whatever but I think it was good that I actually thought I do need to go into hospital because I’m not well and I’m…suicidal and stuff”* (ID 23, Female)

Some participants described diverse self-management strategies to cope with their mental health symptoms, including playing music, white noise, and using technologies such as X-Box and TV. Some participants also mentioned using these technologies as distraction techniques, and to keep their mind busy. Furthermore, participants reported taking avoidance strategies further to “suffer in silence” and “faking” being happy to not burden people, instead carrying on as normal. However, evidently they indicated they became good at hiding their symptoms.

“*It’s almost like I zip up a body bag of everything I’m really feeling and I use the analogy of body bag because obviously they’re dead people*. *And that’s how I feel inside…I cover that up and smartly dressed up*, *makeup on*, *hair*, *comes out and no one knows*, *no one realises… I’m very good at hiding it*. *I’ve become really good at that over the years*.*”* (ID 2, Female)

In contrast to the avoidance strategies, some participants described the unsafe coping strategies they actively used to manage their mental health symptoms. Several participants described activities such as drinking excessively, taking drugs or self-harming to cope with difficult situations. For example, one participant expressed what they needed from self-harm.

*“When I self-harm I need to see the blood to make me feel that I’m either alive or I need to see that I’ve inflicted so much pain on myself that I can bleed*, *you know*. *Scratching doesn’t do anything*, *I need to get a razor and stuff like that”*. (ID 9, Female)

### 2. Signs of mental health deterioration

All participants reported multifactorial changes in either behaviour, mood or cognition as an indication of mental health deterioration and most reported having a combination of all these indicators. Reported behaviours that were identified as signs of mental health deterioration included outbursts, excessive behaviour, social isolation, and changes to routine, eating habits and sleep. Change in sleep patterns (e.g. sleeping too much or not sleeping enough) was the most commonly reported sign of mental health deterioration. For example, most participants mentioned that having trouble sleeping was always an indicator that their mental health was getting worse.

"*Yeah*, *so last year*, *end of January/beginning of February I had a bit of a breakdown because I’d like stayed up not sleeping for like six days because with the mania you don’t feel like sleeping you’re just so inspired by everything and you just want to do stuff”* (ID 20, Female)

*“One of the signs that I now know is an indicator that I should sort of take stick of how things have been going is if I spend all day waiting for it to be time to go to sleep*, *not because I like sleeping but because I want the day to be over”* (ID 29, Female)

Some participants indicated that a clear sign of their mental health deterioration was also change in their mood. For example, irritability, low mood, hopelessness and lack of motivation were all mentioned as signs of deterioration. Participants reported feeling low in mood as one of the first signs indicating their mental health was deteriorating, and some reported mood swings, where they would feel okay one minute, then be crying and feeling hopeless the next. This was particularly the case in participants with bipolar disorder.

“*I wake up one day and I feel really down*, *and I feel really grumpy about everything and I just* … *but it’s not the normal…I can’t be bothered feeling*. *It’s like* … *a different feeling that I can’t really* … *it’s just like a heavy chest feeling like urgh* … *I’ll do a week or two or even potentially longer sometimes feeling crap and then I’ll wake up fine again and I’m like ‘Right*, *that was that done*. *I’m fine*. *Let’s carry on’*.” (ID 14, Female)

In some cases, their low mood escalated quite rapidly and resulted in self-harm and/or suicidality.

“*It’s not even like a hopeless stage*. *It’s just like what is the point*? *You just ask yourself what’s the point*? *What’s the point for even Coco-Pops* … *then I just know it’s going downhill*, *literally*. *Once I say what’s the point for everything*, *yeah*, *it’s just not good”*. (ID 19, Female)

*"I* … *made it to the train station and I* … *went down past the platform and lay down on the tracks there* … *at the time it was to see how I felt about it*, *because I didn’t know* … *I guess like a panic*, *just kind of*, *I just felt the need to do something like I couldn’t just exist in this state”* (ID 30, Male)

A few participants mentioned that signs of deterioration during their manic phases was showing increasing impulsivity, hyperactivity and hearing voices.

### 3. Technology concerns and values

#### Attractive features of technology

Participants reported using technology to “kill time”, engage with others, express themselves and to see positive content. They also reported appreciating the aspects of anonymity and accessibility of technology. For instance, many reported that being able to engage and communicate with other people (anonymously or with named people) outside of routine hours (e.g. Monday–Friday 9am-5pm) and with the almost certainty that someone would respond, made them want to use social media. Moreover, being able to speak openly without judgement about something important to them, particularly in relation to their mental health, was reassuring.

*“[talking to someone online]…you don’t have to deal with the person*. *You’re getting information*, *obviously depending on what type of app it is*, *you're getting information or having an app there*, *but you don’t have to deal with the judgement of another human being*. *Some people feel quite judged and when you’ve got mental health issues that can just heighten everything*, *so … I get why people do shy away from it”* (ID 2, Female)

#### Suitability and contextual features

Most participants described several contextual issues related to using technology to detect deterioration. The main concern was whether deterioration should be captured using an automatic or self-reported process. Some people liked the idea of tracking their mood over time themselves and felt technology (e.g. a mobile app) would be useful to do this. Others felt active participation was necessary for success but that this would be unlikely in people who were unwell because they’d not be in the right mind-set, have motivation or be able to remember how they’d been feeling.

“*If you can get technology which will say something is going wrong with them*, *then yes I mean I think technology is the ideal way to monitor* … [but] *You see*, *the thing about having a mental health disorder is that you are already not mentally healthy*. *Expecting a person who is not mentally healthy to then also monitor themselves and their disorders is a bad idea*” (ID 13, Male)

In contrast, others suggested, instead, to use an automatic process. Monitoring sleep and activity levels over time using wearables, mobile app and monitoring online social media activity (e.g. change of: uploaded content, frequency of postings etc.) was generally deemed feasible and not reliant on the person to self-report. However, several participants were unsure that the latter was possible because of the uncertainty that activity was a genuine cause for concern. For example, two participants described times when they had posted dark images but they would not have deemed themselves unwell or wanting help.

#### Pragmatic concerns

Participants discussed two main pragmatic concerns: safeguarding and viability. Reported safeguarding and validity concerns included being hesitant on using artificial intelligence because of the potential of false negatives (i.e. providing help when it was not needed) or false positives (i.e. not detecting deterioration when it *was* needed and failing to assist). Reported viability issues covered reliance on Wi-Fi, accessibility, expense, losing or stealing the device and battery dying.

“*If that person was in a crisis and then your battery dies or something*, *then they’re by themselves again”* (ID 19, Female)

#### Exposure to and impact of social media

The majority of participants discussed occasions where they had been exposed to negative content on social media including Instagram, Facebook and Tumblr. Reported examples included pro-anorexia (“pro-ana”) forums, pictures and postings; pictures of self-harm; videos showing violence on young people; cyberbullying; instructions on how to kill yourself; and romanticising suicide. Although participants agreed social media content can be harmful to people with mental health difficulties through becoming addicted, having a negative impact on self-esteem and inspiration to imitate unsafe behaviours, some people they knew liked viewing the negative content.

“*People post pictures of their self-harm–really graphic–and I know people who follow them because I can see who they’re following and who’s…liking their pictures*, *and stuff*, *and these people with significant mental health problems*, *I know they’re following almost consciously to make themselves worse*. *People will post all of their pictures of their self-harm or other people’s*, *or just really gory pictures and post about [it] ‘Oh I’ve been sectioned*, *this*, *that and the other*, *as if it’s a good thing*” (ID 1, Female)

“*I want to use social [media] less actually… because although there [are] all these positivity groups and things out there it’s actually just making my mood worse*, *so personally I’ve tried to stay away from social media*.*”* (ID 2, Female)

### 4. Technological applications to detect worsening mental health

The last theme integrates the previous three themes: dealing with mental health symptoms, signs of deterioration (changes in mood, behaviour and cognition) and technological concerns and values, to build them into the possible conceptualisation of a technological intervention. Three steps were identified as important and useful to detect deterioration using some of these technologies: monitoring, flagging warning signs and taking action.

Most participants agreed that monitoring mental health over time using a mobile app, wearable or social media could be possible as it would mean signs of deterioration (e.g. sleep difficulties, low mood) would be spotted early. For example, monitoring social media activity could help flag worsening mental health from not going on it anymore to posting unusual and negative content. One person mentioned flagging physiological changes, such as increase in cortisol, although they were unclear how this would happen practically.

“*Just have some sort of hormone monitoring system*. *I think that would be really great for picking up on someone’s issues*, *because again it is a hormone*, *it is literally the reason why you feel things*. *If you go to the source and start detecting there*, *then there is not that much room for error is there”* (ID 13, Male)

Moreover, many participants felt that whilst monitoring and flagging warning signs was needed, *acting on* the warning signs was paramount. Potential actions reported included: displaying helplines, calling an ambulance or other emergency services, having immediate access to a designated psychiatrist or psychologist online and going to hospital.

*“[have] an emergency button where they’re told*, *“This person is either going to hurt themselves or has hurt themselves”*.*…you need to send a medical professional to where they’re registered to*. *So*, *having the location would probably be … because if you’re not at home and you're having a bad day*, *and they’ve got your location*, *they can come and get you or find or send a medical professional to come and*, *well*, *talk you off the ledge*, *as it were”* (ID 21, Female)

## Discussion

### Key findings and comparison to other studies

This is the first study to examine the perspectives of young people with mental health diagnoses, on the acceptability and feasibility of wearables, social media and other technologies to detect mental health deterioration. The findings identified four broad and interrelated themes: 1) dealing with mental health symptoms, (2) signs of mental health deterioration, (3) technology concerns and values and (4) technological applications to identify worsening mental health.

Wearables and/or mobile apps using regular real-time feedback to detect worsening mood, sleep and/or activity levels as signs of deterioration, delivered through self-report or an automated programme (sleep and activity only) were deemed generally acceptable and feasible to young patients, in line with studies involving adult patients. [20–22,24,29–31] However, monitoring social media activity (changes in: uploaded content, frequency of postings etc.) was considered more problematic mainly because of a weaker association with signs of deterioration.

Despite the generally encouraging response to the use of technology, several concerns would need to be addressed to make future interventions successful. These included: automated or self-reported process, safeguarding issues (false positives and false negatives) and practical issues such as reliance on Wi-Fi, battery and accessibility. Despite these considerations, there is potential for technology to help supplement existing mental health service provision and get people access to help earlier. This is because patients reiterated concerns on the dissatisfaction with current non-technological services, [32,33] particularly in relation to the lack of access, lack of trust and poor engagement with services and in the lead-up to crisis. Our study adds to the growing literature exploring perceptions of acceptability and feasibility in using technology to aid detection, assessment and treatment of mental health difficulties. [9,18]

### Strengths and limitations

There are several strengths to this study. This research was co-produced with young people who have lived experience of mental health difficulties (co-researchers). The co-researchers were directly involved throughout all stages of the research, including data collection, analysis and dissemination. The insight of the co-researchers during the data synthesis and interpretation stages particularly provided a unique lens and helped to ensure the integrity of the data was retained. The triangulation of interpretations between two researchers with expertise in mental health and two co-researchers, resulted in a depth of knowledge and different perspectives, which enhanced data rigour. Similarly, openness, peer debriefing (of both co-researcher and researcher) and recorded reflexivity [34] of all research team members also helped achieve this. All points helped ensure the trustworthiness and accuracy of the self-report data obtained. [35] Another strength of the study was the diversity in participant sample, as participants had a range of diagnoses and co-morbidities. Although participants had a mental health condition they were not in crisis or distress; therefore had capacity to participate and insight into their own experiences both now and when they have experienced a mental health crisis in the past.

There are also limitations. Participants had varied experiences in using different technologies, and some may have had more experience than others; self-reported behaviours could therefore be subject to participant reporting bias. Similarly, researcher bias was possible, as codes may have been influenced by both the researchers’ (e.g. experience in sleep research) and co-researchers experience (e.g. lived experience). Mental health diagnoses varied yet there was an underrepresentation of young people with psychosis and psychotic disorders, with only two participants with this experience recruited. At study inception, it was anticipated that we’d include more participants with experience of schizophrenia and psychosis, however, these people were generally identified as either too unwell to be seen or reluctant to take part in the study. Moreover, only London based, mainly female patients, from one site, were included therefore the transferability [34] is limited.

### Implications

Young patients are at risk of significant deterioration in mental state, hospitalisation or self-harm due to the lack of, or delay in, accessing mental health services. [8,33] The introduction of technologies could help to fill this gap so that those in need are seen and helped earlier. In this study, young patients were diagnosed with a range of different mental health conditions, with most diagnoses being either bipolar disorder or depression. As such, in the first instance, a future technological intervention, such as a wearable or mobile app, to detect deteriorating mental health in young people, may be applicable to these patient groups only. The intervention should be developed to detect worsening sleep, lack of activity and/or low mood in these patient groups which could be a viable first step because most participants identified these as signs of deterioration. Moreover, there is a need for immediate action following detection. Future users should be aware of this and what this will involve to enable them to trust and therefore use the intervention. However, for the intervention to be successful, viability, safeguarding and patient preference should be considered. Indeed, in the first instance, the effectiveness of differing operational platforms (automated or self-report) should be compared to further understand this potential barrier. The intervention should also be co-produced with patients, but industry partners and clinicians may also provide a useful insight to integrate into practice. Moreover, further qualitative research may be needed in some patient groups (patients with schizophrenia or psychosis) to establish specific tailoring that would be acceptable before implementation.

## Supporting information

S1 Table COREQ checklist. (DOC)
